# Three-dimensional Speckle Tracking Echocardiography in Amyloidosis: A New Assessment Method for a Rare Disease

**DOI:** 10.5935/abc.20180182

**Published:** 2018-09

**Authors:** Brivaldo Markman Filho

**Affiliations:** Hospital das Clínicas da Universidade Federal de Pernambuco, Recife, PE - Brazil

**Keywords:** Immunoglobulin Light-Chain Amyloidosis, Cardiomyopathy, Restrictive/psysiopathology, Echocardiography, Three-Dimensional, Heart Failure

Immunoglobulin deposition in the myocardium characterizes involvement of cardiac amyloidosis (CA).^1^ Fibrillary infiltration, which may happen in every heart cavity, leads to the restrictive cardiomyopathy phenotype, with complex pathophysiological mechanisms, which will result in the syndromic diagnosis of congestive heart failure.^2^ Diastolic dysfunction is dominant in most cases, and it may or may not, follow diverse levels of systolic dysfunction in the most advanced phases of the disease. Atrial remodeling by amyloid infiltration may contribute to cardiac output decrease by means of insufficient or nonexistent telediastolic atrial contraction.^3^ The onset of atrial electrical instability, ending in atrial fibrillation, highlights symptomatic worsening and these patients' reserved prognosis.^4^

Historically, the right cavities of the heart have been neglected in echocardiographic assessments. The complex morphology of the right ventricle (RV) has possibly contributed for the lack of reproducible data on echocardiographic cutting plans, diversely from the left ventricle (LV).^5^ The development of three-dimensional echocardiography (3DE) has allowed for a more accurate calculation of right ventricle volume and function in the diverse pathologies involving that chamber.^6^

Regarding the importance of assessing the right atrium (RA), the relation between an increase in its area and adverse clinical outcomes has already been shown.^7-9^ Nevertheless, its asymmetric shape, increased by the occurrence of remodeling, as observed in CA cases, limits a more precise assessment of its volume using two-dimensional echocardiography (2DE).^6^ On the other hand, using three-dimensional echocardiography (3DE) overcomes these limitations, allowing not only for the accurate assessment of right atrium volume changes, but also for the detailed description of its size and function.^9^

In this context, the study by Nemes et al.,^10^ fills the gap regarding the use of 3DE to assess the RA for the diagnosis of CA. The authors noted significant increase in the left atrium diameter, in the interventricular septum thickness and in the LV posterior wall diameter, besides RV systolic dysfunction in patients with light-chain cardiac amyloidosis (LC-CA), when compared to healthy control group patients. These findings, compatible with restrictive cardiomyopathy, have been previously described for CA.^2^ Assessing the RA by three-dimensional Speckle Tracking echocardiography (3DSTE), increased atrial volumes and smaller fractions of total and active atrial emptying in patients with LC-CA were found, when compared to control group patients. Furthermore, according to the authors, findings of reduced values on global strain peak and on segmental area, on circumferential strain in many levels, besides changes in the longitudinal and area strain in atrial contraction, suggest longitudinal e circumferential impairment in RA function in its reservoir and active contraction phases, as well as non-uniform atrial dysfunction. Although the authors have not managed to show differences on the stroke-volume values for RA when compared to healthy control-group patients, they describe the importance of measuring the RA emptying fractions and the strain-values for a proper LC-CA diagnosis.

Kado et al.,^11^ studied longitudinal strain in heart cavities with the purpose of checking if change in a given cavity would have a higher prognostic value than traditional echocardiographic parameters regarding the occurrence of adverse cardiac events. Prognostic relevance was found on strain changes in the four cavities, also the RA longitudinal strain was capable of differentiating LC-CA from non-obstructive hypertrophic cardiomyopathy.

However, the drawing of the study by Nemes et al.^10^ did not permit the conclusion whether the changes described by means of 3DSTE would be LC-CA-specific or if they could be found in another type of infiltrative/restrictive cardiomyopathy. On the other hand, it drives our attention to the need of a more detailed assessment on the right side of the heart, regardless of the underlying disease investigated.

At last, the appearance of innovations on echodopplercardiography, which always occur towards diagnosis improvement or accuracy, as well as to make early LC-CA therapy for the prevention of adverse clinical outcomes, one must not underestimate already established, traditional echocardiographic findings for disease assessment.

## References

[1] Gertz MA, Dispenzieri A, Sher T (2015). Pathophysiology ant treatment of cardiac
amyloidosis. Nat Rev Cardiol.

[2] Mohthy D, Damy T, Cosnay P, Echahidi M, Casset-Senon D, Virot P (2013). Cardiac amyloidosis: updates in diagnosis and
management. Arch Cardiovasc Dis.

[3] Mohty D, Petitalot V, Magne J, Fadel BM, Boulogne C, Rouabhia D (2017). Left atrial function in patients with light chain amyloidosis: a
transthoracic 3D speckle tracking imaging study. J Cardiol.

[4] Park J, Lee SH, Lee JS, Park JH, Joung B, Lee M-H (2016). High recurrence of atrial fibrillation in patients with high
tissue atrial natriuretic peptide and amyloid levels after concomitant maze
and mitral valve surgery. J Cardiol.

[5] Rudski LG, Lai WW, Afilalo J, Hua L, Handschumacher MD, Chandrasekran K (2010). Guidelines for the echocardiographic assessment of the right
heart in adults: a report from the American Society of Echocardiography.
Endorsed by the European Association of Echocardiography, a registered
branch of the European Society of Cardiology, and the Canadian Society of
Echocardiography. J Am Soc Echocardiogr.

[6] Lang RM, Badano LP, Tsang W, Adams DH, Agricola E, Buck T (2012). EAE/ASE recommendations for image acquisition and display using
three-dimensional echocardiography. Eur Heart J Cardiovasc Imaging.

[7] Sallach JA, Tang WHW, Borowski AG, Tong W, Porter T, Martin MG (2009). Right atrial volume index in chronic systolic heart failure and
prognosis. J Am Coll Cardiol Imaging.

[8] Rai ABS, Lima E, Munir F, Khan AF, Waqas A, Bughio S (2015). Speckle tracking echocardiography of the right atrium: the
neglected chamber. Clin Cardiol.

[9] Peluso D, Badano L, Muraru D, Dal Bianco L, Cuccini U, Kocabay G (2013). Right atrial size e function assessed with three- dimensional and
speckle-tracking echocardiography in 200 healthy volunteers. Eur Heart J Cardiovasc Imaging.

[10] Nemes A, Foldeák D, Domsik P, Kalapos A, Kormányos Á, Borbényi Z (2018). Right atrial deformation analysis in cardiac amyloidosis -
results from the three dimensional speckle tracking echocardiographic
MAGIAR-Path Study. Arq Bras Cardiol.

[11] Kado Y, Obokata M, Nagata Y, Ishizu T, Addetia K, Aonuma K (2016). Cumulative burden of myocardial dysfunction in cardiac
amyloidosis assesed using four chamber cardiac Strain. J Am Soc Echocardiogr.

